# Supplementary material for: Construction of an Electrochemical Receptor Sensor Based on Graphene/Thionine for the Sensitive Determination of β-Lactam Antibiotics Content in Milk
Source: Int J Mol Sci. 2020 May 7;21(9):3306. doi: 10.3390/ijms21093306 (PMC7246818; doi:10.3390/ijms21093306)
Supplement: Supplementary file 1 [file ijms-21-03306-s001.pdf]

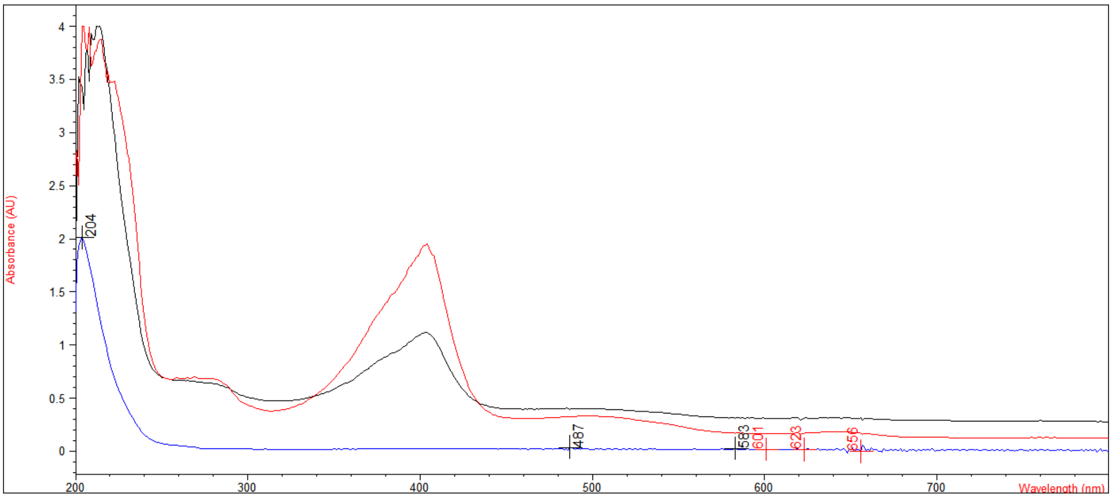


**Figure S1.** UV spectrum of HRP-AMP. The black is the UV spectrum of HRP, the red is the UV spectrum of HRP-AMP and the blue is the UV spectrum of AMP.

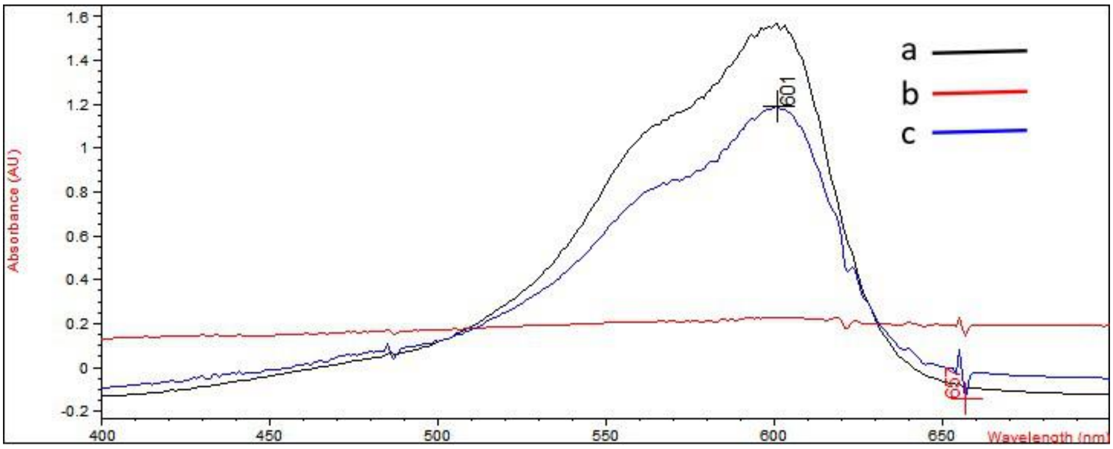


**Figure 2.** UV-vis spectra of TH(a), Gs(b) and GS-TH(c).

**Table S1.** Coefficient of variability of intra-assay and inter-assay.

| Cefquinoxime (μg/L) | Measured value (`X±SD, μg/L) | Intraspecific Coefficient of Variation (CV, n = 5) | Average determination (`X±SD, μg/L) | Coefficient of variation between groups (CV, n = 5) |
|---|---|---|---|---|
| 0.1 | 0.097±0.005 | 5.27 | 0.098±0.003 | 3.06 |
| | 0.099±0.007 | 6.83 | | |
| | 0.097±0.007 | 7.43 | | |
| | 0.101±0.006 | 5.76 | | |
| | 0.094±0.006 | 6.86 | | |
| 0.5 | 0.486±0.030 | 6.24 | 0.495±0.009 | 1.84 |
| | 0.494±0.022 | 4.48 | | |
| | 0.505±0.016 | 3.11 | | |
| | 0.486±0.027 | 5.64 | | |
| | 0.503±0.015 | 3.02 | | |
| 1 | 1.077±0.04 | 3.87 | 1.02±0.047 | 4.61 |

| | | | | |
|---|---|---|---|---|
| | 0.967±0.05 | 4.66 | | |
| | 0.973±0.09 | 9.32 | | |
| | 1.027±0.13 | 12.4 | | |
| | 1.043±0.07 | 7.19 | | |
| 2 | 2.047±0.08 | 3.67 | 2.01±0.034 | 1.69 |
| | 1.977±0.09 | 4.30 | | |
| | 1.991±0.05 | 2.51 | | |
| | 2.040±0.09 | 4.36 | | |
| | 1.980±0.12 | 6.33 | | |
| 4 | 3.907±0.24 | 6.18 | 4.03±0.096 | 2.39 |
| | 4.037±0.18 | 4.50 | | |
| | 4.067±0.24 | 5.95 | | |
| | 3.963±0.26 | 6.59 | | |
| | 4.157±0.12 | 2.81 | | |
| 8 | 8.347±0.28 | 3.35 | 7.98±0.222 | 2.77 |
| | 7.977±0.32 | 3.98 | | |
| | 7.98±0.54 | 6.81 | | |
| | 7.75±0.30 | 3.88 | | |
| | 7.88±0.55 | 6.93 | | |

**Table 2.** Recovery rates and coefficient of variation of β-lactam antibiotic by receptor-sensor in milk sample (n=5).

| medicine | Added (μg/kg) | Recovery (X±SD) | Intraspecific Coefficient of Variation (CV, n=5) | Average recovery (X±SD) | Coefficient of variation between groups (CV, n=15) |
|---|---|---|---|---|---|
| Cefalexin | 40 | 102.89±5.76 | 5.6 | 101.65±3.22 | 3.17 |
| | | 97.99±7.33 | 7.48 | | |
| | | 104.07±8.86 | 8.51 | | |
| | 80 | 95.39±3.64 | 3.81 | 99.86±4.78 | 4.79 |
| | | 99.28±4.41 | 4.44 | | |
| | | 104.90±3.82 | 3.64 | | |
| | 160 | 94.27±4.60 | 4.88 | 100.63±5.59 | 5.56 |
| | | 102.87±6.41 | 6.23 | | |
| | | 104.75±1.87 | 1.78 | | |
| Cefaquinoxime | 10 | 102.11±2.85 | 2.79 | 99.39±2.62 | 2.63 |
| | | 96.89±2.36 | 2.44 | | |
| | | 99.17±3.22 | 3.25 | | |
| | 20 | 97.64±4.75 | 4.87 | 103.07±4.93 | 4.78 |
| | | 104.33±5.37 | 5.15 | | |
| | | 107.25±8.23 | 7.67 | | |
| | 40 | 98.74 ±3.95 | 4 | 102.44±3.89 | 3.8 |
| | | 102.08±2.88 | 2.82 | | |
| | | 106.50±2.94 | 2.76 | | |
| Ceftiofur | 45 | 102.68±5.09 | 4.96 | 100.65±4.24 | 4.21 |
| | | 95.78±3.25 | 3.39 | | |
| | | 103.48±1.82 | 1.76 | | |
| | 90 | 97.43±1.42 | 1.46 | 101.11±3.60 | 3.56 |

| | | | | | |
|---|---|---|---|---|---|
| | | 104.63±2.56 | 2.44 | | |
| | | 101.26±1.84 | 1.82 | | |
| | 180 | 103.86±2.15 | 2.07 | 100.65±2.81 | 2.79 |
| | | 99.47±2.89 | 2.91 | | |
| | | 98.63±4.02 | 4.08 | | |
| Penicillin G | 0.2 | 95.67±10.30 | 10.77 | 92.44±7.38 | 7.98 |
| | | 84.00±4.77 | 5.68 | | |
| | | 97.67±4.25 | 4.35 | | |
| | 2 | 97.17±7.37 | 7.59 | 99.67±4.78 | 4.79 |
| | | 96.67±10.32 | 10.68 | | |
| | | 105.17±11.93 | 11.34 | | |
| | 4 | 91.58±4.47 | 4.89 | 93.81±2.55 | 2.71 |
| | | 96.53±5.97 | 6.18 | | |
| | | 93.25±9.41 | 10.09 | | |
| Ampicillin | 0.5 | 82.73±6.11 | 7.39 | 84.89±2.04 | 2.41 |
| | | 86.80±9.71 | 11.19 | | |
| | | 85.13±4.84 | 5.69 | | |
| | 2 | 97.40±5.96 | 6.12 | 98.68±1.17 | 1.18 |
| | | 98.95±4.35 | 4.4 | | |
| | | 99.68±2.40 | 2.41 | | |
| | 4 | 93.25±1.89 | 2.02 | 95.97±2.36 | 2.46 |
| | | 97.50±5.20 | 5.34 | | |
| | | 97.17±7.35 | 7.56 | | |
